# A light of hope? Inequalities in mental health before and after the peace agreement in Colombia: a decomposition analysis

**DOI:** 10.1186/s12939-021-01381-x

**Published:** 2021-01-19

**Authors:** Sebastián León-Giraldo, Germán Casas, Juan Sebastián Cuervo-Sánchez, Catalina González-Uribe, Antonio Olmos, Noemi Kreif, Marc Suhrcke, Oscar Bernal, Rodrigo Moreno-Serra

**Affiliations:** 1 Alberto Lleras Camargo School of Government, Universidad de los Andes, Bogotá, Colombia; 2 School of Medicine, Universidad de los Andes, Bogotá, Colombia; 3 Centre for Health Economics, University of York, York, UK

**Keywords:** Conflict, Mental health, Inequalities, Colombia, Peace accord

## Abstract

**Background:**

The present study seeks to evaluate the change in mental health inequalities in the department of Meta after the signing of Colombia’s Peace Agreement in 2016 with the FARC guerrilla group. Using a validated survey instrument composed of 20 questions (‘SRQ-20’), we measure changes in mental health inequalities from 2014, before the signing of the agreement, to 2018, after the signing. We then decompose the changes in inequalities to establish which socioeconomic factors explain differences in mental health inequalities over time.

**Methods:**

Our study uses information from the *Conflicto, Salud y Paz* (CONPAS) survey conducted in the department of Meta, Colombia, in 1309 households in 2018, with retrospective information for 2014. To measure inequalities, we calculate the concentration indices for both years. Through the Oaxaca change decomposition method, we disaggregate changes in mental health inequalities into its underlying factors. This method allows us to explain the relationship between changes in mental health inequalities and changes in inequalities in several sociodemographic factors. It also identifies the extent to which these factors help explain the changes in mental health inequalities.

**Results:**

Mental health inequalities in Meta were reduced almost by half from 2014 to 2018. In 2018, the population at the lower and middle socioeconomic levels had fewer chances of experiencing mental health disorders in comparison to 2014. The reduction in mental health differences is mostly attributed to reductions in the influence of certain sociodemographic variables, such as residence in rural zones and conflict-affected territories, working in the informal sector, or experiencing internal displacement. However, even though mental health inequalities have diminished, overall mental health outcomes have worsened in these years.

**Conclusions:**

The reduction in the contribution of conflict-related variables for explaining mental health inequalities could mean that the negative consequences of conflict on mental health have started to diminish in the short run after the peace agreement. Nevertheless, conflict and the presence of other socioeconomic inequalities still contribute to persistent adverse mental health outcomes in the overall population. Thus, public policy should be oriented towards improving mental health care services in these territories, given the post-accord context.

## Background

Colombia’s civil armed conflict has lasted more than 50 years and, by recent counts, has caused approximately 262,197 deaths, 80,154 forced disappearances, 15,687 victims of sexual assault, and almost 7,305,936 internally displaced people [1]. After 4 years of peace negotiations, Colombia’s government, and *Fuerzas Armadas Revolucionarias de Colombia* (FARC-EP), one of the most significant and influential guerilla groups in Colombia’s armed conflict, signed a peace agreement on November 24, 2016, entitled *General Agreement for the End of the Conflict and the Construction of a Stable and Durable Peace*. This agreement led to the demobilization of the FARC group and its incorporation as a political party in Colombia. Even though the agreement does not represent the end of conflict-related violence, the existence of a peace treaty is intended to facilitate public policy work in conflict-affected territories, specifically in places where the presence of the government was problematic due to the dominance of the FARC group.

Arguably, one of the most invisible consequences of the armed conflict has been its impact on mental well-being [2]. Numerous studies have measured the impact that Colombia’s civil conflict has generated on the mental health of populations affected by armed conflict. Studies have mostly emphasized the consequences on population groups historically affected by direct conflict violence, such as displaced populations and militaries [3], demobilized guerilla groups [4], or vulnerable populations such as women and children [5]. Even though the peace process contributed to the de-escalation of direct conflict violence in Colombia, less is known about its short-run impacts on conflict-affected territories; in particular, the mental well-being of people historically affected by the armed struggle has not been sufficiently explored.

Colombia has long been considered one of the most unequal societies in Latin America [6]. There are considerable regional differences in socioeconomic status, particularly within territories deeply affected by armed conflict. One of these territories is the department of Meta, located in the Eastern Plains region proximate to the Andes mountain range at the center of the country. Municipalities in this territory have been exposed to different levels of the Colombian armed conflict [7]. These differences in conflict incidence, along with socioeconomic inequalities between territories and households, have generated enduring mental health inequalities [8].

These inequalities, in conjunction with political instability, may in turn increase the chances of civil conflict and limit economic development and recovery [9]. Sustained mental health inequalities limit development potential in a region as a consequence of reduced productivity and well-being [10]. Simultaneously, these inequalities broaden differences between social groups due to limited physical, psychological, and social resources for improving their living conditions [11].

Few studies worldwide have explored the evolution of mental health inequalities in conflict-affected territories. National and international research has mostly focused on measuring the relationship between mental health outcomes and conflict de-escalation in postconflict scenarios. Roberts, Damandu, Lomoro, and Sondorp [12] have found a high prevalence of PTSD symptoms (36%) and depression (50%) in victims of armed conflict after Sudan’s Peace Agreement. Nevertheless, their study does not evaluate mental health outcomes before the signing of the agreement, and hence does not allow identifying changes in mental health outcomes over time. Other recent national studies [13] analyze mental health outcomes in postconflict Colombia, but as they are focused on qualitative reported experiences, they do not allow for inference as to whether, for the overall population, mental health outcomes have improved after the agreement and whether inequalities in mental health have diminished.

Against this background, the present study seeks to evaluate the change in mental health inequalities from 2014 to 2018 in the department of Meta, a territory that has been heavily affected by armed conflict. First, we measure mental health inequalities in both years and describe changes over time. We then use a change decomposition method to establish whether variations in these inequalities over time can be explained by changes in the distribution of important socioeconomic factors between groups and/or by a reduction in the determinants’ explanatory power. Finally, we evaluate whether changes in mental health inequalities have been associated with improvements in overall mental health outcomes for the inhabitants of conflict-affected territories. In the following sections, we describe our methodological approach, present our results, and offer a discussion of the main conclusions driven by our analysis.

## Methods

Our study uses information from the *Conflicto, Paz y Salud* (CONPAS) survey conducted in 1309 households of the department of Meta. The survey is representative at the level of conflict incidence of the municipalities. It was conducted in 2018 and includes retrospective information for 2014.

Our survey sample was selected through a probabilistic design, stratified at the level of conflict incidence of the municipality and urban and rural territories. Through a multi-stage sampling, block areas were selected and, within these blocks, one household. To select the sampling units, a Simple Random Sampling without Replacement method was conducted.

The tendency to present mental health disorders was measured using the Self-Report Questionnaire (SRQ-20) [14], an instrument developed by the World Health Organization (WHO) that is composed of 20 questions regarding general health and well-being in the last month. If a person answers *yes* to 8 or more of the 20 questions of the questionnaire, he/she is considered to present a positive tendency towards experiencing mental health disorders [14]. In this paper, we refer to the case of a person responding *yes* to 8 or more questions of the SRQ-20 questionnaire as SRQ+ (i.e., a SRQ positive case).

We constructed the Household Wealth Index (HWI) [15], that measures socio-economic status capturing information about access to various assets. This index will be used throughout the analysis to capture inequalities in the distribution of mental health disorders. The HWI is defined by Equation (1) as follows:

1 $$ \mathrm{HWI}={\upalpha}_1\left(\frac{{\mathrm{x}}_1-{\overline{\mathrm{x}}}_1}{{\mathrm{s}}_1}\right)+{\upalpha}_2\left(\frac{{\mathrm{x}}_2-\overline{{\mathrm{x}}_2}}{{\mathrm{s}}_2}\right)+\dots {\upalpha}_{\mathrm{k}}\left(\frac{{\mathrm{x}}_{\mathrm{k}}-\overline{{\mathrm{x}}_{\mathrm{k}}}}{{\mathrm{s}}_{\mathrm{k}}}\right) $$

where x_j_ is a variable that measures access to a specific household asset related to wealth (e.g., home appliances.), ${\overline{\mathrm{x}}}_{\mathrm{j}}$ is the mean of this variable, s_j_ its standard deviation and α_j_ is a specific weight for the variable obtained through Principal Components Analysis (PCA), using the first component of the PCA as an estimator. The index is constructed through the weighted summation of *k* variables that measure access to different household assets. We calculated an HWI indicator for both 2014 and 2018 independently to determine the concentration indices.

Along with the SRQ and the HWI indicator, we estimated health concentration indices (HCI) [16] using the SRQ indicator for 2014 and 2018. The concentration index measures the distribution of SRQ+ cases at different socioeconomic levels. The method orders all individuals from the poorest to the richest using the HWI indicator as a classification variable. Then, the number of SRQ+ cases is classified in each specific socioeconomic group. Both variables are plotted in a graph as cumulative distributions. In the no-inequality scenario, the graph presents an even distribution of SRQ+ cases. For example, 20% of the population should represent 20% of the total SRQ+ cases. This hypothetical scenario represents the perfect equality curve. The concentration index is the ratio between the concentration curve and the perfect equality line, as shown in Eq. 2:

2 $$ HCI=\frac{2\ \mathit{\operatorname{cov}}\left(Y,R\right)}{\mu_y} $$

where Y is the health variable (SRQ+), *μ*_*y*_ is its mean, and R is the person’s rank (or position) in the income distribution.

The concentration index offers some advantages compared to alternative methods to measure health inequalities. Firstly, the concentration index allows us to estimate inequalities in health across the entire socioeconomic distribution and, simultaneously, analyze the extent to which these inequalities originate from inequalities in other socioeconomic determinants. Secondly, it allows us to overcome some limitations of frequency ratios or relative risk measures (e.g., odds ratios). In particular, as the concentration index uses a continuous socioeconomic variable, it avoids the issue of establishing cut-off levels to define strata. Moreover, odds ratios tend to overestimate the size of the relationship between variables, when the occurrence of the health event is higher than 0.20%, and, similar to other frequency-related measures, usually are sensitive to the size of comparison groups. This poses limitations for the use of odds ratios for cross-comparisons between years or when there are differences in the size of comparison groups and the outcome variables [17].

The concentration index ranges from − 1 to 1, with − 1 being absolute inequality favoring the rich, 0 perfect equality, and 1 perfect inequality favoring the poor. Nevertheless, as SRQ is a binary outcome variable, Wagstaff [18] proposed a mathematical correction to adjust the HCI to adequate ranges (Eq. 3):

3 $$ H{CI}_N=\frac{HCI}{1-{\mu}_y\ } $$

With this transformation, the range of our HCI moves from *μ*_*y*_ − 1 to 1 − *μ*_*y*_, ensuring that the HCI can be interpreted between the values of a standard concentration index.

The data cleaning and preparation process is described in appendix 1. All calculations were conducted at the individual level, relevant unit for the survey and analysis, as responses for the SRQ questionnaire were only administered to the head of the household. This approach also allows us to analyze how individual sociodemographic characteristics contribute to the increase/decrease of health inequalities over time. To enable comparisons over time, we use the responses of people who – during the entire four-year period of our study – were adults (i.e., 18 years or older) and lived in Meta, resulting in sub-samples of 1309 adults for year 2018 (the actual year of the survey) and 1089 adults in year 2014. This sample restriction allows us to focus on analyzing changes in health inequalities in a single territory and overcome other confounding factors related to mental health issues in minors.

To explain changes in mental health inequalities, we selected a group of variables that have been found in the international literature to be associated with individual mental health outcomes in conflict-affected regions. Specifically, we included (*i*) demographic and health-related variables [19, 20]: age of the respondent, gender, zone of residence (urban or rural), ethnicity and an assessment of health functioning using the World Health Organization Disability Assessment Schedule (WHODAS); (*ii*) socioeconomic variables [21]: type of work (formal, informal) and education level; and (*iii*) conflict-related variables [22]: a measure of conflict incidence in the municipality where the household is located, and whether the respondent is previously or currently experiencing internal displacement. Using a probit model, we calculated marginal effects to estimate the influence of each independent variable x on the probability of SRQ+ cases. The influence of these independent variables for explaining mental health outcomes can be expressed using Eq. 4.

4 $$ {\eta}_x={\beta}_X\frac{\mu_x}{\mu_y\ } $$

Where *μ*_*x*_ and *μ*_*y*_ are, respectively, the means of the independent and dependent variables. Eq. 4 measures the relative importance that variable x has on explaining the mean of the SRQ variable, using the marginal effects of the Probit model.

Through the Blinder- Oaxaca decomposition method [23], we decomposed the change in the health concentration index from 2014 to 2018 into two components using Equations (5) and (6):

5 $$ \Delta  HCI={\sum}_{\boldsymbol{x}}{\eta}_{x2018}\left({C}_{x2018}-{C}_{x2014}\right)+{\sum}_{\boldsymbol{x}}{C}_{x2014}\left({\eta}_{x2018}-{\eta}_{x2014}\right)+\Delta  \left(\frac{G{C}_{\varepsilon t}}{\mu_t}\right) $$

6 $$ \Delta  HCI={\sum}_{\boldsymbol{x}}{\eta}_{x2014}\left({C}_{x2018}-{C}_{x2014}\right)+{\sum}_{\boldsymbol{x}}{C}_{x2018}\left({\eta}_{x2018}-{\eta}_{x2014}\right)+\Delta  \left(\frac{G{C}_{\varepsilon t}}{\mu_t}\right) $$

Both equations are alternative ways of decomposing the change in HCI using different weighting variables. Through an estimation of individual concentration indices *C* for each independent variable x or for all x combined (∑_***x***_), this method allows us to analyze to which extent changes in health inequalities are attributable to changes in inequality in the determinants of health (*C*_*x*2018_ − *C*_*x*2014_) rather than to changes in their elasticities (*η*_*x*2018_ − *η*_*x*2014_) [18]. These total effects, expressed independently in the two summation components, may reinforce themselves or have opposite effects. The last component, $\Delta  \left(\frac{G{C}_{\varepsilon t}}{\mu_t}\right)$ for *t* = 2014 or 2018, denotes the change in a generalized concentration index (obtained as a residual term) reflecting the change in the HCI that cannot be explained by the first two terms in Equations (5) and (6). Either equation allows us to establish which sociodemographic factors contribute to the increase /decrease of mental health inequalities over time. We estimated both models along with the concentration indices and the decompositions of the HCI for both years to evaluate changes in mental health inequalities and establish which factors explain these changes over time.

## Results

### Mental health inequalities in 2014 and 2018

**Fig. 1:**
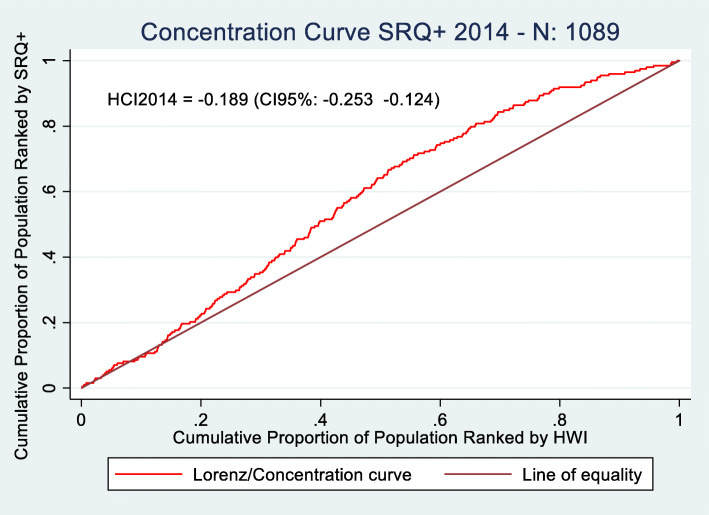
Concentration Curves SRQ+ 2014 (N:1089). Source: Prepared by authors based on CONPAS 2014 and 2018. *P*-value HCI2014 = 0.000. P-value HCI2018 = 0.000

**Fig. 2:**
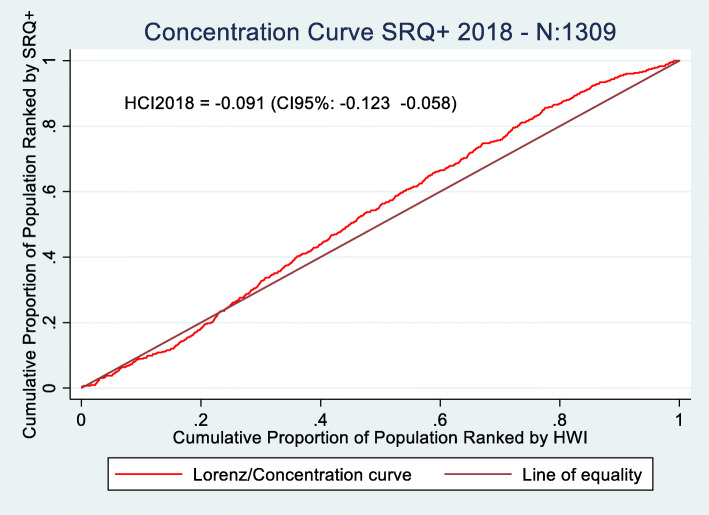
Concentration Curves SRQ+ 2018 (N:1309). Source: Prepared by authors based on CONPAS 2014 and 2018. *P*-value HCI2014 = 0.000. P-value HCI2018 = 0.000

Figures 1 and 2 show the concentration curves for the distribution of SRQ positive (SRQ+) cases among different income levels of the population.

The negative coefficient of the concentration indices indicates that there is an unequal distribution of SRQ positive cases in both years among the population of Meta. For 2014 and 2018, the population at lower socioeconomic levels have a greater tendency to present mental health disorders than those at higher income levels. Those in middle socioeconomic groups have a higher incidence of SRQ+ cases for both years compared to lower and higher socioeconomic groups. A higher proportion of SRQ cases is found in people with incomes in the range between 40 to 60%. Nevertheless, in 2018 there is a significant reduction in SRQ inequality measured through concentration indices, with a change of − 0.189 to − 0.091. The difference in concentration indices between groups (2014 and 2018) is significant at the 95% confidence level (*p*-value: 0.048).

By analyzing the behavior of both curves, it is possible to understand how tendencies to present mental health disorders have changed over time. The first noticeable change can be found in the range between 0 and 20%. The 45-degree line, the perfect equality level, shows the expected accumulated number of SRQ+ cases at each income level. Unlike 2014, in 2018 people in the lowest socioeconomic levels (0–20%) have fewer SRQ+ cases than expected, as can be observed in Fig. 2 from the drop in SRQ+ cases below the perfect equality line in the 10–20% range. Inequalities in the tendency to present mental health disorders start to appear, in 2018, at the 30% income level, where the number of SRQ+ cases surpasses the perfect equality line (Fig. 2). Nevertheless, compared to 2014 (Fig. 1), inequalities in the middle-income groups are growing at a lower rate, as shown by the smaller gap between the number of accumulated SRQ+ cases (red curve) and the perfect equality line in Fig. 2. Even though there are inequalities in the distribution of SRQ+ cases in both years, these differences are lower in 2018 compared to 2014.

### Oaxaca - blinder change decomposition

Changes in mental health inequalities may be explained by changes in inequalities in socioeconomic variables that are correlated to mental health outcomes. The influence of these determinants can be expressed through two different mechanisms. The first involves changes in the importance of specific socioeconomic variables in explaining the mental health concentration index, regarded as the elasticities or unexplained components. The second mechanism involves changes in the magnitude of inequalities in determinant factors, measured by the concentration indices of each socioeconomic determinant. Table 1 presents a disaggregation of both mechanisms using the specifications of the Oaxaca- Blinder method in Eqs. 5 and 6. For both equations, we calculate the weighted change in inequalities for each socioeconomic determinant (*ΔCη*) as well as the influence of these determinants over time (*ΔηC*). We then estimate the total change in the concentration index of each socioeconomic variable, adding the two previous components (*Total*). Finally, we calculate how much of the change in mental health inequalities may be attributed to changes in each determinant (*%*).

**Table 1:** Decomposition of the Change in the Concentration Index for Mental Health Disorders (2014–18)

	Decomposition of change in concentration index - Oaxaca type approach							
	Equation (5)		Equation (6)		Total		Total	
	ΔCη	ΔηC	ΔCη	ΔηC	Total1	%	Total2	%
*Displaced*								
No	Baseline							
Si	0.005**	0.007	0.006**	0.006	0.012	8%	0.013	9%
*Age group*								
18–44 years	Baseline							
45–64 years	0.000	−0.010	− 0.001	− 0.009	− 0.010	−7%	− 0.010	−7%
65 or more	0.000	− 0.002**	0.001	− 0.003^**^	−0.002	−1%	− 0.002	− 1%
*Sex*								
Male	Baseline							
Female	0.007	0.007**	0.006	0.009**	0.014	10%	0.015	10%
*Zone of residence*								
Urban	Baseline							
Rural	0.007*	0.015	0.004	0.018	0.022	15%	0.023	16%
*Work type*								
Formal	Baseline							
Informal	−0.008	0.067	0.005	0.053	0.059	42%	0.059	41%
Out of labor force	0.002	−0.002	0.001	−0.001	0.000	0%	−0.000	0%
*Conflict level*								
Capital city	Baseline							
Highly affected	0.001	0.021*	0.000	0.022*	0.022	15%	0.022	15%
No conflict	−0.000	− 0.012	0.000	− 0.013	− 0.012	−8%	− 0.012	− 8%
Lowly affected	− 0.003	0.037*	0.007	0.026*	0.034	24%	0.034	23%
*Ethnicity*								
*Majority*	Baseline							
*Minoriy*	0.000	−0.000	−0.000	− 0.000	0.000	0%	−0.000	0%
*Education*								
None	−0.000	0.001	0.001	0.001	0.001	1%	0.001	1%
Primary School	0.004	−0.015	−0.014	−0.011	− 0.011	−8%	−0.011	−8%
Secondary School	−0.001*	0.011	0.008*	0.010	0.010	7%	0.010	7%
Undergraduate	Baseline							
*WHODAS*	0.010	−0.007**	−0.005	0.003**	0.003	2%	0.003	2%
Residual								
Total	**0.024**	**0.118**	**0.019**	**0.111**	**0.142**		**0.145**	

Source: Own analysis based on CONPAS 2014 and 2018

Abbreviations: *WHODAS* World Health Organization Disability Assessment Schedule

(Note: *p-value<=0.5, **p-value<=0.01)

The results presented in Table 1 allow us to assess to what extent changes in mental health inequalities over time are caused by changes in the distribution of an explanatory factor along different socioeconomic groups (column ΔCη) or changes in the elasticities of the associations between these explanatory factors and the chances of experiencing mental health disorders (column ΔηC). If these values are negative, both effects reinforce one another and contribute to increasing the inequality gap. If both are positive, both effects are contributing to a reduction in the inequality gap.

The percentage column at the end of Table 1 indicates which variables contribute the most to these changes in mental health inequalities, where the variables with positive values represent those with a higher contribution. For the most critical determinants - sex, the zone of residence, conflict affectation, displacement, and education level – most of the consequent reduction in inequalities in mental health is a consequence of changes in elasticities, rather than changes in the relative distribution of these factors among different socioeconomic groups, as it is observed from the larger elasticity effects (column ΔηC) in comparison to the distribution effect (column ΔCη) for these variables.

Differences between the results estimated using equations (5) and (6) are virtually inexistent, indicating that both Oaxaca decomposition specifications lead to similar results. The generally small magnitude of the estimated coefficients in Table 1 is intuitive, to the extent that significant changes in the distribution of sociodemographic characteristics among populations of different poverty levels are not expected in this short period. The results show that displacement status, sex, conflict intensity of the place of residence and working status contributed to a reduction in the mental health inequality gap (positive coefficients) during these 4 years, especially in conflict-affected territories. Living in a territory slightly or highly affected by conflict has much less influence in explaining mental health inequalities, given its positive coefficients of 0.022 and 0.025 for the elasticity component of the change in concentration index. There is also a lower influence of being employed informally (0.053) and living in rural zones (0.018) for explaining inequalities over time.

The change in the concentration index of displacement and sex are both a consequence of changes in their relative importance (elasticity) for explaining mental health inequalities as well as changes in the distribution of these variables (women and displaced people) across different income groups. This means that both variables reduce mental health inequalities because of the reduced importance of these variables in determining mental health inequalities and a relatively more equitable distribution of these populations (e.g., displaced, and non-displaced individuals) across different income levels.

Even though mental health inequalities may have been reduced over time, it is also important to assess if the overall incidence of mental health disorders in the population has improved or not. The concentration indices only evaluate relative distribution, but not the changes in absolute numbers of SRQ cases over time. Table 2 shows the number of SRQ+ cases for both years disaggregated by poverty quintiles, not to establish associations between these two variables, but to analyze the magnitude of possible mental health disorders (SRQ+) at different poverty levels.

**Table 2:** Incidence of SRQ+ cases 2014–2018 by poverty quintiles

***SRQ +***	***SRQ+ 2014 (N = 1089)***		***SRQ+ 2018 (N = 1309)***	
	***Cases***	***Percent***	***Cases***	***Percent***
***Quintile 1***	39	23.1	66	18.6
***Quintile 2***	50	29.6	94	26.5
***Quintile 3***	38	22.5	77	21.7
***Quintile 4***	28	16.6	70	19.7
***Quintile 5***	14	8.3	47	13.3

Source:Prepared by authors based on CONPAS 2014 and 2018

Table 2 shows that the number of people with possible mental health disorder (SQR+) increased in all poverty quintiles. Also, the distribution of people with possible mental health disorders (SQR+) has changed between poverty quintiles. Quintile 1 passed from 23.1 to 18.6% of total SRQ+ cases, and quintile 2 moved from 29.6 to 26.5%. Quintile 3 maintained a similar behavior over time in the SRQ+ distribution, with a change of only 0.8 percentage points over this period (22.5 to 21.7%). Nevertheless, for the high-income groups, SRQ+ cases increased from 16.6 to 19.7% (Quintile 4) and from 8.3 to 13.3% (Quintile 5).

These results show that overall inequality has diminished mostly because of a relatively higher tendency to present mental health disorders among people at higher income levels compared to lower socioeconomic groups. However, the likelihood of experiencing mental health disorders increased for all population groups between 2014 and 2018.

## Discussion

### Main conclusions

Results show that mental health inequalities decreased by almost half between 2014 and 2018, after the signing of the peace agreement in 2016. Nevertheless, the number of SRQ+ cases also increased simultaneously across all income levels. Differences among sex, rural and urban zones, participation in the informal sector, and differences among territories of various conflict incidence levels are the primary determinant factors in explaining mental health inequalities in both years.

Changes in inequalities from 2014 to 2018 are attributed to a more equitable distribution of SRQ+ cases. In 2018, people at higher socioeconomic levels had higher chances of experiencing mental health disorders compared to those at lower socioeconomic levels. These changes in mental health inequalities are partially explained by a lower influence of several determinant factors on mental health inequalities, specifically, conflict-related variables, such as internal displacement and conflict incidence.

Reduction in mental health inequalities, and especially, the lower influence of conflict-related variables, may reflect the short-run positive consequences of conflict de-escalation, which usually have a more direct impact on people exposed to everyday violence. People at lower income levels are often exposed more intensely to conflict-related violence as a consequence of insecurity in the regions or territories where they live. Reductions in direct conflict violence may improve mental health outcomes on these populations, revealing, simultaneously, other risk factors that may be more prominent in higher-income groups (work stress, greater responsibilities and so on). Nevertheless, this new sociopolitical scenario represents an adequate context for increasing awareness and treating mental health disorders in these communities.

However, results also show that, even in this scenario, negative mental health outcomes persist, not only in conflict-affected territories but also in other populations and civilian groups. These continuous adverse mental health outcomes may be a consequence of the persistent long-run trauma and negative experiences that years of conflict violence have on people’s well-being and health. These factors require time as well as health services to improve in the long run. Meta, as well as several other territories in Colombia, still suffer from conflict-related violence such as threats and assassinations of social leaders by small factions, dissident groups of the demobilized FARC, and other guerilla groups that are still active. Therefore, results emphasize the importance of providing mental health services for the overall population to improve well-being and diminish the psychological impact of conflict-related events over time.

Even though we partially analyze mechanisms that explain the reduction of mental health inequalities in the short run, our results leave some open questions and hypotheses about the relationship between social determinants and mental health inequalities. Although conflict is an important factor for explaining mental health outcomes, the increment in tendencies to present mental health disorders between 2014 to 2018 shows that persistent socioeconomic differences still contribute and perpetuate health inequities even after conflict de-escalation. People living under these circumstances still have important challenges and limitations accentuated after the conflict, such as searching for jobs, resuming education, rebuilding social ties and relationships, and restoring confidence among peers and institutions. All of these situations are negatively affected during armed conflict and have an impact on well-being. More importantly, results show that mental disorders may easily transcend socioeconomic groups and impact even the most accommodated and economically stable individuals in conflict scenarios. The psychological consequences of conflict are difficult to overcome. For this reason, mental health services are required for extended time periods, and, most importantly, consistently, and holistically appropriate to the different circumstances that impact mental health well-being and are exacerbated by conflict.

Increases in poor mental health, especially in middle and high-income groups, are challenging to interpret. To an extent, such increases are a manifestation of how armed conflict transcends different socioeconomic groups and levels and is not exclusive to the most vulnerable populations. In some cases, middle-income groups may have disadvantages in accessing mental health services. For example, middle-income groups may experience difficulties for accessing health services that are prioritized and subsidized for the lower socioeconomic groups in the public healthcare system, due to their relatively better economic situation. At the same time, they may be unable to pay for the better-quality health services that high-income groups can afford, particularly in the private healthcare sector. This social level’ trap’, commonly referred to as the *missing middle* [24], may be especially crucial in these vulnerable contexts, but further analyses are necessary to arrive at definitive conclusions.

### Comparison with previous international and national studies

Few international studies have evaluated the evolution of mental health outcomes and inequalities in conflict-affected territories. Most of them have found a high prevalence of mental health disorders in postconflict societies but have not reviewed its relationship with health inequalities along time. Roberts, Damandu, Lomoro, and Sondorp [12] analyze mental health outcomes after the peace agreement of the Sudan civil conflict in 2005 through a survey of 1242 respondents in Juba, the capital city of South Sudan. The study finds a high prevalence of mental health disorders 4 years after conflict de-escalation, mostly related to conflict exposure and the long-run effects of trauma. However, studies of the influence of socioeconomic circumstances and inequalities are limited, and the analysis focuses on demographic characteristics without a more in-depth analysis of socioeconomic variables. De Jong, Komproe, Van Ommeren [25] review the short-run psychological consequences of armed conflict along time on 3048 respondents in Algeria, Cambodia, Ethiopia, and Palestine, finding a high prevalence of PTSD symptoms and anxiety disorders. Nevertheless, their conclusion focuses only on the influence of direct conflict violence in population groups.

In Colombia, the studies evaluating changes in mental health outcomes have also been limited. Burgess and Fonseca [26] evaluate mental health distress in displaced people among 40 victims of internal displacement. In this study, the mental health burden in postconflict scenarios is highly related not only with past violence but sustained poverty and social inequality, low paid work, unemployment, and low support from government services. Nevertheless, its conclusions are mainly focused on conflict victims, not overall population groups, and they do not evaluate changes over time. Cuartas et al. [8] measure mental health inequalities using the National Mental Health Survey and identify critical determinants for health differences in conflict affected regions. However, their study concentrates on a specific point of time (2015) without evaluating changes over time in conflict-affected territories.

We can nevertheless draw comparisons with the results found by Cuartas et al. [8] as their study also uses concentration indices for quantifying mental health inequalities related to conflict in Colombia, specifically, at a country level, for 2015. Cuartas et al. [8] estimate a mental health concentration index of − 0.12, showing that mental health disorders are unevenly distributed in Colombia in the overall population, being less pronounced in comparison to Meta in 2014 where these inequalities are much more significant (− 0.189). These results show that mental health inequalities may be more critical in regions in Colombia where conflict has been more rampant and severe across time – territories that usually are also characterized by higher socioeconomic inequalities. Therefore, our results show preliminary exploratory results of the importance of guaranteeing adequate access to mental health services, especially during periods of armed struggle.

Mental health inequality levels in Meta diminished to an HCI value of − 0.9 in 2018. This leaves some open questions, specifically, whether during the same period, national mental health inequalities also diminished. If this is the case, it is crucial to assess whether mental health inequalities could have been reduced to a larger extent in conflict-affected territories than national levels. Our results could signal that conflict is a major contributor to persistent mental health inequalities in war zones.

To the best of our knowledge, no previous studies in Colombia have assessed mental health inequalities in non-conflict affected territories using concentration indices. Nevertheless, we may draw benchmarks from other international studies, which have calculated health inequalities in non-conflict territories to analyze how severe mental health inequalities may be in conflict regions. Morasae et al. [27] estimated mental health inequalities in Iran’s capital, Tehran, finding a concentration index of − 0.067. Mangalore et al. [28] estimates mental health inequalities in an adult population in the UK in 2007 finding a concentration index of − 0.079. In both cases, inequality is mainly driven by avoidable socioeconomic inequalities. However, concentration indexes are much smaller than those found in our study, both before Colombia’s Peace Accord (CI: − 0.189) and after it (CI:-0.091), showing how conflict may be a major driver that exacerbates health inequalities.

### Strengths and weaknesses

Our key contribution is our analysis of mental health outcomes in conflict-affected territories in the short-run after Colombia’s Peace agreement and the change in mental health inequalities over time. This study, more than judging the success or failure of this peace treaty or assessing causality in changes in mental health outcomes, seeks to provide a clearer perspective of current health trends in these territories to improve policy-oriented decisions and help identify key health areas to focus on in postconflict scenarios.

Moreover, our study is based on a large-scale survey that allows us to analyze mental health outcomes in Colombia more broadly among war victims and overall population groups, with a focus on a territory with particularly high conflict incidence levels in certain municipalities comparison to other regions of Colombia. This analysis is not common in most literature about mental health in Colombia and in international literature. Our study contributes to further discussions of the short-run consequences of peace agreements in conflict-affected territories.

Besides our contribution to the literature on health inequalities and conflict, our study offers further insights into the relationship between socioeconomic inequalities and mental health. It offers a critical perspective of how peace agreements and conflict de-escalation do not necessarily translate into immediate improvements in mental health outcomes. The existence of socioeconomic inequalities may limit the positive effect of conflict de-escalation over time. Our analysis offers some critical perspectives on the mental health effects of short-run peace agreements in economies characterized by persistent socioeconomic differences.

The use of retrospective information may introduce recall bias in some analyses, due to difficulties experienced by respondents in recalling certain events, feelings, or moods. To reduce recall bias, our questionnaires were designed, and enumerators trained to ask the participant, before conducting the 2014 questions, to remember a personal or family event which occurred in that year, using, as a memory stimulating story, a noteworthy event that occurred in 2014 and that was easily discernible for the majority of the respondents (Colombia’s national football team participation in the 2014 FIFA World Cup). Even though some recall bias may still persist in this scenario, information directly reported by individuals is often the best – and sometimes the only available – source of information in areas where, like conflict-affected regions, health reporting systems may not operate adequately to provide information that is sufficiently robust in terms of quality, or where conducting data collection exercises is infeasible due to security concerns (as in many areas of 2014 Meta).

Even though we found reductions in mental health inequalities in the years we analyzed, our data does not allow us to locate specific moments or years at which these inequalities started to diminish. In this sense, inequalities could have begun to decrease even before the signing of the Peace Agreement. Our study has concentrated on the analysis of a specific territory in Colombia; as such, some of our conclusions may be limited to the social circumstances of this area. Nevertheless, we believe that the analysis of a territory that has been historically affected by the conflict, with varying levels of conflict intensity observed across its municipalities, may provide valuable insights for the understanding of changes in health outcomes (and the formulation of relevant public policies) in other conflict-affected settings with comparable characteristics.

## Conclusions

Mental health inequalities in conflict-affected zones may broaden social inequalities and limit social and economic development. Even though civil conflict persists in some regions, Colombia’s peace treaty has reduced direct conflict violence and created a better environment for peace promotion and improvement of physical and psychological health. De-escalation of conflict has diminished the effects and influence that war-related events have on mental health. Still, the conflict has led to persistent mental health differences and increased chances of experiencing mental health disorders. A reduction of the influence of war on mental health outcomes offers opportunities for broadening social health policies oriented to health recovery and promotion in war-torn communities.

The increment of SRQ positive cases in all population groups shows that, even though the conflict incidence and internal displacement are less determinant in explaining health inequalities, the overall population is still affected by several socioeconomic circumstances and difficulties that have been adversely affecting mental health. Results show the influence that context, aside from individual circumstances, has on social and health outcomes over time, commonly known as neighborhood effects [29]. Living in vulnerable social circumstances is likely to maintain adverse health outcomes even with a reduction of conflict. This is particularly true in territories where political and social instability remains fragile, and where years of conflict violence have diminished coping mechanisms and public policy support. Conflict, through its impact in society, may also disrupt daily routines (sleeping, adequate nourishing etc.) in vulnerable populations, important for maintaining overall wellbeing, consequences that previous research has shown may partially explain associations between low socioeconomic status and mental health outcomes [30].

Public policies designed to promote capacity building, economic and social development are indispensable for promoting long-run improvements in health and, simultaneously, quality of life among conflict-affected populations. In the short term, these policies may focus on strategies for improving mental healthcare in these territories. In the long run, policies can focus on strengthening healthcare systems, access, and quality of health services, not only oriented to conflict victims but to the overall population, to improve mental health outcomes in these communities.

## Figures and Tables

**Fig. 3:**
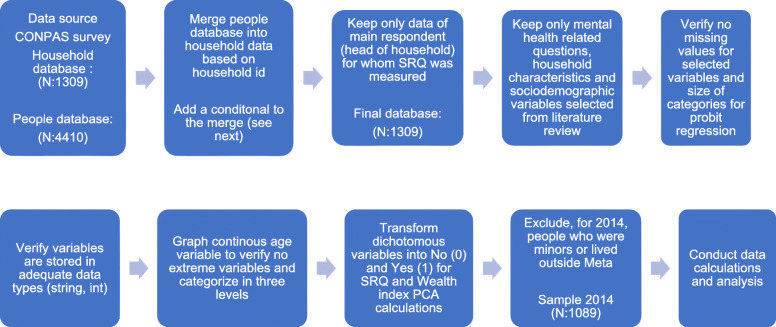
Description of the data cleaning procedure. Source: Prepared by authors

## Data Availability

The datasets used and/or analyzed during the current study are available from the corresponding author on reasonable request.

## Appendix 1

The flow diagram in Fig. 3 describes the process carried out to construct the final database used for this analysis.

Data collection was conducted by a specialized survey company from Colombia using the sample design described in the methods section. The database has information about the main respondent and their family members. We kept only the main respondent’s information (in addition to household-level information), as mental health questions were only administered to this person. All variables for the estimation, except age, were categorical or dichotomous and no missing values were found in the responses. To guarantee balanced and precise probit estimations in the decomposition model, the category size of the independent variable was tabulated, finding no bias towards a specific response or categories with low frequency rates (less than 30 cases), procedure recommended before conducting data estimations [31]. The age variable was categorized into three levels following Cuartas et al. [8] approach to favor comparisons with that study. Finally, one last data verification was conducted to make sure all variables were stored as an adequate data type (string, integers etc.)

The responses referring to dichotomous variables, initially in a 1 (no) and 2 (yes) scale, were transformed into a 0 (no) 1 (yes) to conduct PCA analysis and to calculate the SRQ score to measure positive tendency to have a mental health disorder, following the criteria recommended by the World Health Organization [14]. To estimate the Wealth index for 2014, we excluded data for people who were minors in 2014 or lived outside of the Meta department, filtering out people who reported a household address outside of the Meta department or who were minors in 2014. These decisions were due to comparability issues, to minimize the risk of comparing people who may have been impacted by external events in other areas of the country beyond Meta (conflict-related or not), and to eliminate concerns about differences in mental health-related outcomes (and their drivers) between minors and adults.
